# Multiple detection of pathogens in ticks: development of a high throughput real time PCR chip used as a new epidemiologic investigative tool

**DOI:** 10.1186/1756-3305-7-S1-O12

**Published:** 2014-04-01

**Authors:** L Michelet, S Delannoy, E Devillers, G Umhang, A Aspan, M Juremalm, J Chirico, FJ Van der Wal, H Sprong, TP Boye Pihl, K Klitgaard, R Bødker, P Fach, S Moutailler

**Affiliations:** 1 USC BIPAR, Animal Health Laboratory, ANSES, Maisons-Alfort, France; 2 IdentyPath Platform, Food Safety Laboratory, ANSES, Maisons-Alfort, France; 3 Nancy Laboratory for Rabies and Wildlife, Wildlife EcoEPIdemiology & Surveillance Unit, ANSES, Malzéville, France; 4 Department of Bacteriology, National Veterinary Institute (SVA), Uppsala, Sweden; 5 Department of Virology, Immunobiology and Parasitology, National Veterinary Institute (SVA), Uppsala, Sweden; 6 Department of Infection Biology, Central Veterinary Institute, Wageningen UR, Lelystad, the Netherlands; 7 Laboratory for Zoonoses and Environmental Microbiology, National Institute for Public Health and Environment (RIVM), Bilthoven, the Netherlands; 8 National Veterinary Institute, DTU, Copenhagen, Denmark

## 

Worldwide, ticks transmit more pathogens than other arthropods. Around 60 bacteria, 30 parasites and 100 viruses have been registered as tick-borne pathogens; a third of these pathogens are responsible for zoonoses. Usually, detection of tick-borne pathogens depends on the tick species collected: assays are performed for a restricted number of pathogens that are known to be transmitted by a particular tick species collected at a particular site. To better understand the epidemiology of tick-borne pathogens, it will be important to detect for each sample (one tick or one pool of ticks) most of the diseases they potentially transmit, regardless of the tick species. The aim is therefore to develop a new epidemiologic investigative tool which could detect high number of tick-borne pathogens by real time PCR.

We developed a chip (BioMark™ dynamic arrays, Fluidigm Corporation) targeting pathogens of worldwide distribution transmitted by ticks. The designed epidemiologic arrays may detect 48 pathogens in 48 samples corresponding to 2304 qPCR reactions on the same time. Specific primers and probe have been designed for each pathogen and their specificity have been tested in silico with Blast. To begin, we targeted: (i) 37 pathogens whose *Francisella tularensis*, *Coxiella burnetii*, *Neoehrlichia mikurensis*, 5 species of *Anaplasma*, 3 species of *Ehrlichia*, 8 species of *Borrelia*, 2 species of *Bartonella*, 4 species of *Rickettsia*, 10 species of *Babesia *and 2 species of *Theileria*, (ii) 5 species of ticks whose 3 species of *Ixodes *and 2 species of *Dermacentor*. Sensitivity of primers and probe has been tested on a dilution range of reference DNAs of the targeted pathogens on a Lightcycler 480. Specificity then has been tested on a Biomark™dynamic array. The chip was secondly evaluated on field samples corresponding to 47 pools of 25 nymphs collected in two sites in France, the Netherlands and Denmark (corresponding to 7050 nymphs in total). We succesfully detected and determined the prevalence of *Anaplasma phagocytophilum*, *Neoehrlichia mikurensis*, *Rickettsia helvetica*, *Bartonella henselae*, five different genospecies of *Borrelia burgdorferi *s.l., the recently identified pathogen *Borrelia miyamotoi*, and two parasite species *Babesia divergens *and *Babesia venatorum*. This fast and low-cost tool allows comprehensive testing of tick-borne pathogens and can be customized to fit regional demands or to accommodate new or emerging pathogens. The tool represents a major improvement for surveillance and future epidemiological studies.

